# Anaphylatoxin C5a modulates hepatic stellate cell migration

**DOI:** 10.1186/1755-1536-7-9

**Published:** 2014-05-30

**Authors:** Dola Das, Mark A Barnes, Laura E Nagy

**Affiliations:** 1 Center for Liver Disease Research, Department of Pathobiology, Lerner Research Institute, Cleveland Clinic Foundation, Cleveland, Ohio, USA; 2 Department of Gastroenterology, Lerner Research Institute, Cleveland Clinic Foundation, Cleveland, Ohio, USA; 3 Department of Molecular Medicine, Case Western Reserve University, Cleveland, Ohio, USA

**Keywords:** Complement, Fibrosis, Chemotaxis, Wound healing

## Abstract

**Background:**

C5a and its cognate receptor, C5a receptor (C5aR), key elements of complement, are critical modulators of liver immunity and fibrosis. However, the molecular mechanism for the cross talk between complement and liver fibrosis is not well understood. C5a is a potent chemokine regulating migration of cells in the innate immune system. Since activation and migration of hepatic stellate cells (HSC) are hallmarks of liver fibrosis, we hypothesized that C5a contributes to fibrosis by regulating HSC activation and/or migration.

**Results:**

Primary cultures of mouse HSC increased expression of alpha smooth muscle actin (α-SMA) and collagen 1A (Col1A1) mRNA in response to activation on plastic. Expression of mRNA for C5aR, but not C5L2, a second C5a receptor that acts as a negative regulator, increased in parallel with markers of HSC activation in culture. Increased expression of C5aR on activated HSC was confirmed by immunocytochemistry. Cell surface expression of C5aR was also detected by flow cytometry on activated HSC isolated from mice expressing GFP under the control of the collagen promoter after exposure to chronic carbon tetrachloride. To understand the functional significance of C5aR expression in HSC, we next investigated whether C5a influenced HSC activation and/or migration. Challenge of HSC with C5a during culture had no effect on expression of α-SMA and Col1A1, suggesting that C5a did not influence HSC activation. Another important characteristic of HSC is their migratory capacity; migration of HSC in response to platelet derived growth factor (PDGF) and monocyte chemoattractant protein-1 (MCP-1) has been well characterized. Challenge of HSC with C5a enhanced HSC migration almost as efficiently as PDGF in a two-dimensional wound healing and Boyden chamber migration assays. C5a also stimulated expression of MCP-1. C5a-induced cell migration was slowed, but not completely inhibited, in presence of 227016, a MCP-1 receptor antagonist, suggesting C5a-induced migration occurs via both MCP-1-dependent and -independent mechanisms.

**Conclusions:**

These data reveal that C5a regulates migration of HSC and suggest a novel mechanism by which complement contributes to hepatic fibrosis. C5a and its receptors are therefore potential therapeutic targets for the prevention and/or treatment of liver fibrosis.

## Background

Hepatic stellate cells (HSC), also known as Ito cells or lipocytes, are the major cell type contributing to liver fibrosis. HSC constitute about 2% to 6% of the total cell population in the liver and are located in the space of Disse, between hepatocytes and sinusoidal endothelial cells. In a healthy liver, HSC primarily function in the homeostasis of vitamin A [1]. However, upon activation under conditions of liver injury, HSC lose their vitamin A deposits and transform into a myofibroblast-like phenotype, characterized by the expression α-smooth muscle actin (α-SMA) and collagen [2]. Activation of HSC is a hallmark of the wound healing response leading to accumulation of extracellular matrix, scar formation, and fibrosis [3,4]. Interestingly, there is a growing body of evidence that HSC exhibit a number of characteristics typical of cells in the innate immune system, including active phagocytosis [5,6], expression of Toll-like receptors [7], and components of the inflammasome [8]. However, our understanding of the function for these innate immune pathways in HSC and the development of fibrosis is still incomplete.

Complement, comprised of a large number of distinct plasma proteins, as well as membrane-bound receptors and regulatory proteins, is a critical component of the innate immune system that provides links to adaptive immunity [9]. Proteolysis of C5 is one of the key events of complement activation, generating the small cleavage fragment, C5a. C5a, an anaphylatoxin, induces or amplifies multiple innate immune responses. C5a exerts most of its functions through interaction with its cognate receptor, C5a receptor (C5aR) [10]. However, an emerging body of evidence indicates the involvement of a second C5a receptor, C5L2, which acts as a negative modulator of C5aR activity [11,12]. C5aR and C5L2 share significant homology in structure [13] and are expressed on macrophages, dendritic cells, and neutrophils [14]. C5a binding to C5aR induces rapid auto-phosphorylation, internalization, and subsequent signalling leading to acute inflammatory responses and chemotaxis [11,12].

Recent studies have identified complement as an important contributor to a variety of liver disorders such as viral hepatitis, alcoholic liver disease, non-alcoholic steatohepatitis, and liver fibrosis [15]. C5 is identified as a quantitative trait gene in mouse and human that contributes to hepatic fibrosis across species [16]. However, the molecular and cellular mechanisms by which C5 contributes to fibrosis have not been well studied. Complement is dynamically activated in response to carbon tetrachloride (CCl_4_)-induced liver fibrosis [17]. Complement activation products generated in response to fibrotic insults are known to be involved in both the priming of hepatocytes for regeneration and the clearance of cellular debris. However, much less is known about the possible role of C5a and its cognate receptors in the regulation of HSC. In one report, challenge of LX2 cells, an immortalized human stellate cell line, with C5a increased expression of α-SMA and collagen [18].

Here we have investigated the interaction of complement with primary cultures of murine HSC, interrogating the ability of C5a to mediate activation and migration of HSC. We find that C5a receptors, C5aR and C5L2, are expressed upon HSC activation both in culture and *in vivo*. Challenge of primary cultures of murine HSC with C5a did not influence their activation state; however, C5a potently stimulated migration of HSC. Taken together these data suggest that interaction of C5a and its receptor are important contributors to the migration of activated HSC and thus contribute to the progression of hepatic fibrosis.

## Results

### Expression of mRNA for complement receptors during activation of primary HSC in culture

**Figure 1:**
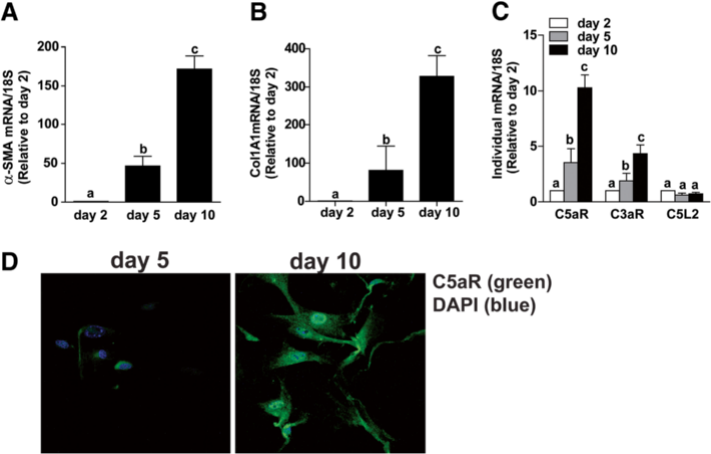
**Activation of HSC induced expression of C3aR and C5aR, but not C5L2.** Primary HSC were isolated from C57BL/6 mice and grown in culture. Expression of **(A)** α-SMA and **(B)** collagen1A1 and **(C)** C3aR, C5aR, and C5L2 mRNA was measured by qRT-PCR and normalized to 18S. Values represent means ± SEM, N = 5. **(A/B)** Values with different superscripts are significantly different from each other (*P* <0.05). **(C)** *P <0.05 compared to day 2. **(D)** Confocal immunocytochemistry for C5aR protein expression was assessed in HSC on day 5 and 10 of culture. C5aR (green) and DAPI (blue). Images are representative of three independent HSC isolations.

Activation of primary HSC during culture is associated with increased expression of α-SMA (Figure 1A) and collagen1A1 (Col1A1) (Figure 1B) [19]. If complement activation products are important regulators of HSC function in the context of hepatic fibrosis, then HSC should express complement receptors. While expression of C3aR, C5aR, and C5L2 was low in quiescent HSC, expression of mRNA for both C3aR and C5aR, but not C5L2, increased in parallel with α-SMA and Col1A1 mRNA from day 2 to day 10 in culture (Figure 1C). Increased expression of immunoreactive C5aR, assessed by confocal imaging, was detected during HSC activation from day 5 to day 10 (Figure 1D), paralleling the rise in C5aR mRNA expression during HSC activation.

### Activated HSC isolated from mice treated with CCl_4_ express C5aR and C5L2

**Figure 2:**
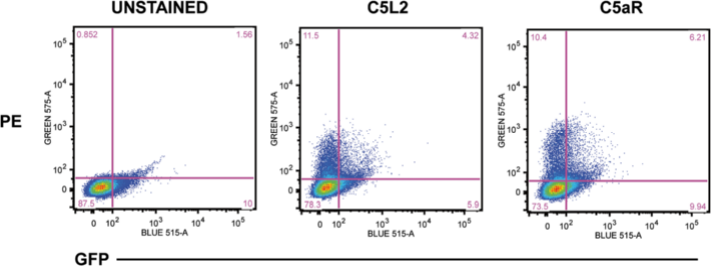
**CCl**_**4**_**-induced fibrosis was associated with expression of C5aR/C5L2 on activated HSC.** Transgenic mice expressing GFP under the control of the collagen promoter were exposed to CCl_4_ for 5 weeks to generate fibrosis. Total NPC were isolated and analyzed by flow cytometry. Cells were stained with anti-C5aR and anti-C5L2 (PE) and gated for GFP and PE. Images are representative of three independent HSC isolations.

While the phenotype of primary HSC activated in culture typically models the phenotype of HSC activated in the liver in response to fibrotic insult, it is critical to validate results of cell culture experiments with data from HSC activated *in vivo*. To check expression of C5a receptors in HSC activated *in vivo*, transgenic mice expressing GFP under the regulation of the collagen promoter [20] were injected with CCl_4_ twice weekly over a period of 5 weeks to induce fibrosis. Flow cytometric analysis was performed on the total non-parenchymal cell population isolated from these mice. The population of cells positive for GFP-fluorescent protein, indicating an activated, collagen-producing phenotype, also expressed C5aR and C5L2 on their cell surface (Figure 2).

### C5a did not enhance the activation of primary HSC in culture

**Figure 3:**
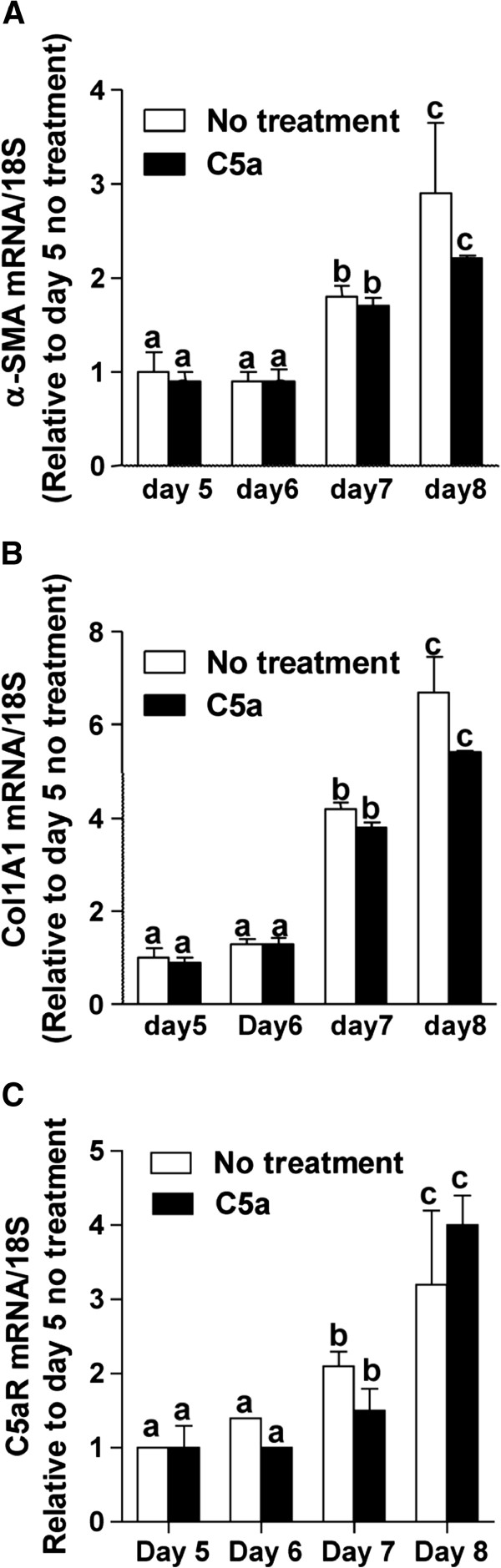
**C5a did not affect HSC activation or expression of C5aR and C5L2.** Mid-quiescent cells (day 5 in culture) were treated or not with C5a (10 ng/mL). Expression of **(A)** α-SMA, **(B)** Collagen1A1 and **(C)** C5aR mRNA was measured by qRT-PCR and normalized to 18S. Values represent means ± SEM, N = 4. Values with different superscripts are significantly different from each other (*P* <0.05).

To understand if C5a contributed to the activation of primary HSC during culture, mid-quiescent primary HSC (day 5) were treated with or without 10 ng/mL C5a until day 8. While culture from days 5 to 8 increased expression of α-SMA and Col1A1 mRNA, addition of C5a did not influence expression of these markers of HSC activation (Figure 3A and B). Further, treatment of HSC with C5a had no effect on the expression of either C5aR (Figure 3C) or C5L2 (data not shown) mRNA.

### C5a enhanced HSC migration in two-dimensional wound healing and Boyden chamber cell migration assays

**Figure 4:**
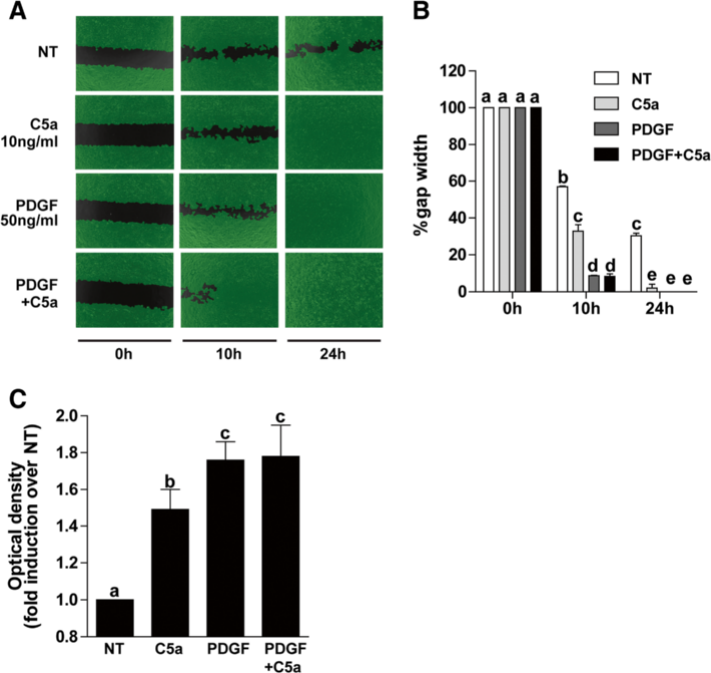
**C5a enhanced HSC chemotaxis.** HSC on day 8 of culture were used in a two-dimensional wound healing assay **(A, B)**. Cells were treated or not with C5a (10 ng/mL) and/or PDGF (50 ng/mL). Images were captured at 0 h, 10 h, and 24 h. Gap width and rate of wound healing were analyzed with Wimasis image analysis software. **(C)** Boyden chamber chemotaxis: On day 8 of culture, HSC were serum starved for 18 h, then detached and resuspended in serum-free DMEM. Inserts containing cells were immersed in serum-free DMEM with or without 10 ng/mL C5a, 50 ng/mL PDGF or both. Chemotaxis was assessed after 10 h incubation, as described in Methods. Values represent means ± SEM, N = 3 for wound healing assay, N = 5 for boyden chamber chemotaxis assay. Values with different superscripts are significantly different from each other.

Upon activation, HSC synthesize chemokines that facilitate migration of infiltrating immune cells, as well as additional HSC, into the site of injury [21]. Since C5a exhibits chemotactic properties [22], we tested whether C5a modulated migration of activated HSC in a two-dimensional wound healing assay. The effect of C5a on HSC migration was compared to that mediated by platelet derived growth factor (PDGF), a potent modulator of HSC migration. Wound closure in untreated cells reached 40% to 60% by 10 h and 24 h, respectively, after injury (Figure 4A, B). Treatment of HSC with 10 ng/mL C5a increased the rate of wound healing both at 10 h and 24 h, with wound healing completed by 24 h after injury. Cells treated with 50 ng/mL PDGF exhibited rapid wound healing, with complete cellular coverage of the injury by 10 h (Figure 4A, B). PDGF and C5a, under the conditions of this experiment, did not have an additive effect. Similarly, even when lower concentrations of C5a (5 ng/mL) and PDGF (25 ng/mL) were tested, PDGF and C5a did not have additive effects on migration (data not shown).Results from the two-dimensional wound healing assay were then confirmed in a standard Boyden chamber migration assay. HSC migration was increased by 1.5-fold upon treatment with 10 ng/mL C5a (Figure 4C). PDGF treatment stimulated HSC migration approximately two-fold and, as in the two-dimensional assay, the chemotactic activity of C5a and PDGF were not additive under these conditions (Figure 4C).

### Role for MCP-1 in C5a-stimulated migration in response to injury in primary HSC

**Figure 5:**
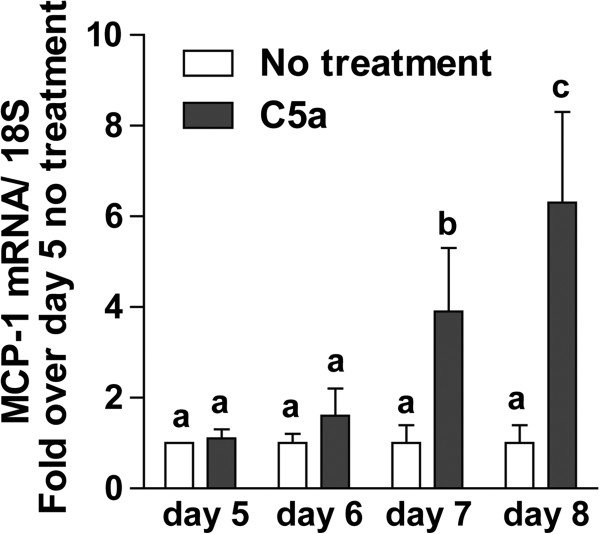
**C5a induces MCP-1 mRNA in HSC.** Primary HSC on day 5 of culture were treated or not with C5a (10 ng/mL). MCP-1 mRNA level was measured by qRT-PCR and normalized to 18S. Values represent means ± SEM, N = 4. Values with different superscripts are significantly different from each other.

**Figure 6:**
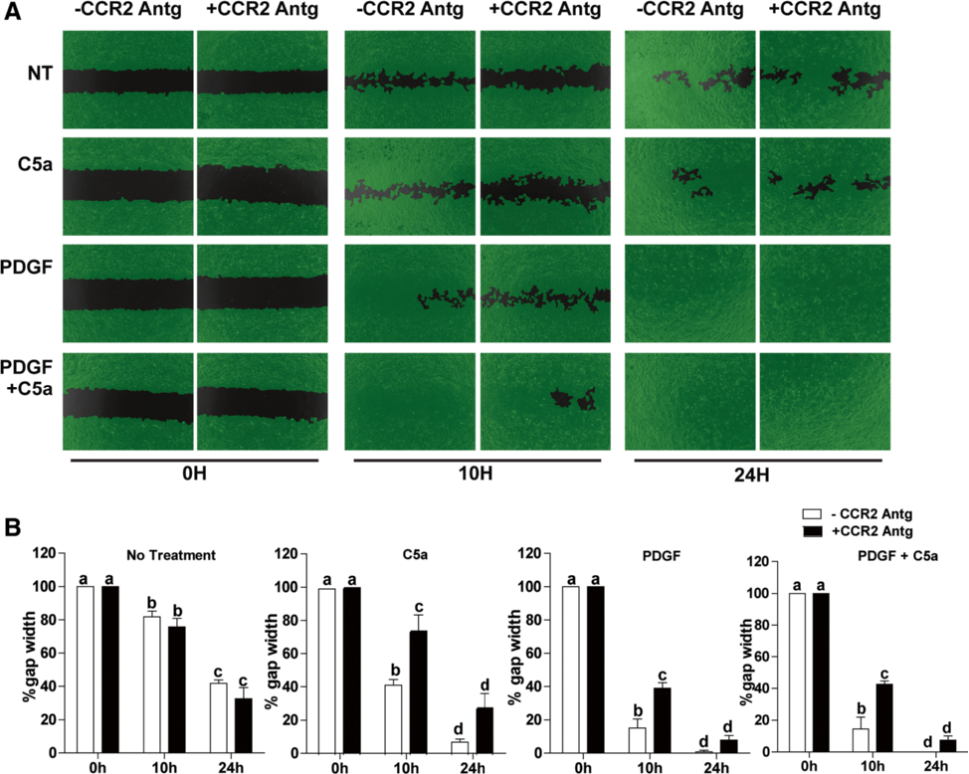
**Antagonism of the MCP-1 receptor (CCR2) slowed C5a mediated HSC migration.** HSC on day 8 of culture were used in a two-dimensional wound healing assay **(A,B)**. Cells were pre-treated with or without a CCR2 receptor antagonist, 227016 (10 nM), for 30 min and then challenged with C5a (10 ng/mL) and/or PDGF (50 ng/mL). Images were captured at 0 h, 10 h, and 24 h following stimulation. Gap width was analyzed by Wimasis image analysis software. Values represent means ± SEM, N = 4. Values with different superscripts are significantly different from each other.

C5a has direct and potent chemotactic activity, but in other cell types, such as macrophages, C5a also increases the expression of MCP-1, another potent chemokine [23]. Consistent with this previous data in macrophages, when primary mid-quiescent HSC (day 5 in culture) were stimulated with C5a for 72 h, expression of MCP-1 mRNA increased by six-fold (Figure 5). Based on these data, we hypothesized that C5a may enhance the migration of HSC in response to injury via direct and/or indirect mechanisms. To understand the role of MCP-1 in C5a-mediated chemotaxis, a CCR2 (MCP-1 receptor) antagonist (227016) was used in the wound healing assay. While the CCR2 antagonist had no effect on the rate of wound healing in non-treated HSC, it delayed the wound healing response in cells treated with C5a or PDGF (Figure 6A,B). MCP-1 receptor antagonist decreased the rate of C5a-mediated cell migration; even after 24 h, complete healing did not occur. The rate of cell migration was also slowed in cells treated with PDGF in presence of CCR2 antagonist; however, PDGF-treated cells were completely restored by 24 h even in the presence of antagonist (Figure 6).

## Discussion

Complement, a critical component of the innate immune system, has been implicated in the progression of a variety of liver diseases, particularly related to hepatic fibrosis [15]. Importantly, genetic studies have identified a role for C5 in fibrotic responses in multiple tissues, in both humans and mice [16]. However, the mechanism by which C5 contributes to fibrosis is not well studied. Here we identified for the first time that activated primary HSC express both C5aR and C5L2. C5aR expression was increased both at the level of mRNA and protein. While C5a did not influence the activation of primary HSC during culture, C5a was a potent chemotactic stimulus to activated HSC, assessed in both two-dimensional wound healing and Boyden chamber cell migration assays. The chemotactic activity of C5a on HSC was due, at least in part, to an increase in the expression of MCP-1, another chemokine that potently stimulates HSC migration. Importantly, these data point to the potential for both C5aR and chemokine receptor antagonists, such as the CCR2 antagonist 227016, as therapeutic agents for the prevention and/or treatment of fibrosis.

C5a acts primarily through its receptor C5aR. C5aR belongs to the G-protein coupled receptor (GPCR) family and is expressed in a wide range of cell and tissue types including brain, liver, and adipocytes. C5aR comprises of seven trans-membrane domain and upon binding to C5a results in activation of the heterotrimeric guanine nucleotide-binding proteins or G-proteins, which ultimately leads to signalling via cyclic adenosine monophosphate (cAMP) and calcium mitogen-activated protein (MAP) kinases [12]. C5L2 or GPR77 is the second receptor for C5a also reported to have seven transmembrane domains but uncoupled from G-proteins [24]. C5aR and C5L2 are expressed by inflammatory cells, dendritic cells, macrophages, lymphocytes, and monocytes, as well as a wide range of cells in liver, lung, kidney, and heart, under specific physiological conditions [11,12]. Here we report for the first time that activated HSC express both C5aR and C5L2; expression of C5aR, but not C5L2, increased in activated compared to quiescent HSC. There is growing appreciation that HSC express multiple receptors and pathways that are typically considered to be part of innate immune responses, including expression of TLRs and inflammasome components [7,8]. These data add a sensitivity to complement to this growing list of innate immune competencies expressed by HSC.

Activation of HSC is regulated by a complex yet coordinated set of transcription factors, including NFκB, AP-1, C/EBP, PPAR, Kruppel like transcription factors and E-box transcription factors [25]. C5a receptor signals via multiple pathways, including cAMP, MAPKs, and PI3K/AKT-dependent signalling [12]. In macrophages, C5a also stimulates the activation of C/EBPβ and C/EBPδ [26]. Given the complex signalling events initiated with C5a agonism, we first tested the ability of C5a to increase activation of HSC in culture on plastic, as well as the regulation of expression of Col1A1 mRNA. C5a did not modulate the activation of primary cultures of HSC, nor increase Col1A1 expression (Figure 1). This is similar to a previous report that C5a induced fibronectin in primary rat HSC but failed to induce collagen or α-SMA [27], but contrasts with another study that reported C5a increased both α-SMA and collagen in LX2 cells, an immortalized human stellate cell line [18]. The difference between these data is likely due to the difference in activation stimulus provided by culture of primary HSC on plastic [28] as compared to the mid-quiescent like phenotypic stability of the LX2 cell line.

While C5a did not modulate activation of primary HSC culture on plastic, it did have a potent stimulatory effect on HSC migration in a two-dimensional wound healing assay and in the Boyden chamber cell migration assay. This response is consistent with the strong chemotactic activity of C5a on other cell types [12]. The impact of C5a on HSC migration was nearly as robust as PDGF, known to be a potent stimulus for HSC migration. MCP-1 is another important chemokine which is upregulated upon HSC activation and implicated in the progression of hepatic fibrosis. C5a induces MCP-1 expression in a number of cell types [29-31]; stimulation of MCP-1 expression, as well as other cytokines and chemokines, is considered to be a mechanism by which C5a amplifies inflammatory responses. While the pathway by which C5a increases MCP-1 expression is not well understood, it is dependent on C5a receptor-mediated activation of AKT in RAW 264.7 macrophages [23] and is mediated at a pre-translational level in other cells of the innate immune system [29]. Consistent with these previous reports of C5a-stimulated MCP-1 expression in other cell types, C5a also induced expression of MCP-1 mRNA in activated HSC. Taken together, these data demonstrate that C5a has potent chemotactic effects on HSC that are both dependent and independent of MCP-1. During the fibrotic response in the liver, recruitment of immune cells to the site of injury is equally as important as the recruitment of HSC. Since C5a has chemoattractant effects on both immune cells [12] and HSC (Figure 4), it is likely that C5a contributes via multiple pathways to the progression of hepatic fibrosis.

## Conclusions

Taken together, these data demonstrate for the first time that the anaphylatoxin C5a modulates migration of HSC, a key cell type involved in hepatic fibrosis. This work links two important aspects in the generation and progression of fibrosis, complement, and HSC. HSC, activated both in culture and *in vivo*, expressed both C5aR and C5L2; expression of C5aR increased with activation of HSC in culture. Although activation of primary HSC was not affected in response to challenge with C5a, HSC migration was enhanced in the presence of C5a. C5a also induced expression of MCP-1 mRNA in HSC and antagonism of the MCP-1 receptor (CCR2) partially inhibited HSC migration. These data add complement to the growing list of innate immune activities that are observed in activated HSC and provide insights into possible therapeutic regulation of complement in the prevention and/or treatment of liver fibrosis.

## Methods

### Materials

Female C57BL/6 mice were purchased from Jackson Laboratory. Recombinant Mouse Complement Component (C5a) was procured from R & D Systems (Minneapolis, MN, USA). Cell culture reagents were from Invitrogen (Grand Island, NY, USA). Hank’s balanced salt solution (HBSS) and PBS were purchased from Media Core (CCF, Cleveland, OH, USA). Common reagents were from Sigma Aldrich unless mentioned otherwise.

### Hepatic stellate cell isolation and culture

All procedures involving animals were approved by the Cleveland Clinic Institutional Animal Care and Use Committee. Adult mice (aged 20 to 36 weeks old) were anesthetized and livers from three mice finely minced in a sterile petri-dish. Minced liver was enzymatically processed in Hank’s balanced salt solution (HBSS) containing pronase (7.5 mg/mL) and collagenase (2 mg/mL) for 30 m at 37°C. Liver digests were then centrifuged at 50 g for 2 m to pellet hepatocytes. Supernatant was collected and centrifuged for 7 m at 1,500 g to collect non-parenchymal cells (NPC). Total NPCs were then re-suspended in HBBS containing DNaseI (0.26 mg/mL) and centrifuged for 6 m at 1,500 g. Hepatic stellate cells were purified from the total NPCs by centrifugation on a histodenz gradient [32]. HSC were checked for retinyl ester deposits in presence of UV light on day 2 of culture; approximately 70% to 80% of cells contained retinyl ester deposits. Purified HSC was cultured in DMEM media, supplemented with 10% heat inactivated FBS and 1% Penicillin-streptomycin-fungizone (PSF). Cells from day 2, day 5, and day 10 in culture were used to isolate RNA and synthesize cDNA. To understand the effect of C5a on HSC activation and complement receptor expression, day 5 HSC were treated or not with C5a (10 ng/mL in HSC culture media) and cultured for 72 h.

### Quantitative real-time PCR

Total RNA was isolated using RNeasy micro kit (Qiagen) according to manufacturer’s instructions. RNA (200 ng) was reverse transcribed using the RETRO script kit (Ambion). qRT-PCR was performed in an MX3000p (Stratagene, La Jolla, CA, USA) using Brilliant II SYBR Green qRT-PCR master mix (Cat# 600828, Agilent Technologies, USA). Primers used for qRT-PCR was synthesized by Integrated DNA Technologies (Coralville, IA, USA). Details of all the primer sequences used are provided in Table 1. Statistical analysis of qRT-PCR data was performed using ΔCT values.

**Table 1:** qRT-PCR primer sequence

α-SMA	Forward- 5′GTCCCAGACATCAGGGAGTAA3′
	Reverse - 5′TCGGATACTTCAGCGTCAGGA3′
Col1A1	Forward - 5′ATGTTCAGCTTTGTGGACCTC3′
	Reverse - 5′CAGAAAGCACAGCACTCGC3′
C5aR	Forward - 5′GTGGGTTTTGTGTTGCCTCT3′
	Reverse - 5′TGATAGGGCAGCCAGAAGAT3′
C3aR	Forward - 5′ TCGATGCTGACACCAATTCAA3′
	Reverse - 5′ TCCCAATAGACAAGTGAGACCAA3′
C5L2	Forward - 5′ACCACCAGCGAGTATTATGACT3′
	Reverse - 5′GCTGCATACAGCACAAGCA3′
MCP-1	Forward - 5′AGGTCCCTGTCATGCTTCTG3′
	Reverse - 5′TCTGGACCCATTCCTTCTTG3′
18S (rat/mouse)	Forward - 5′ACGGAAGGGCACCACCAGGA3′
	Reverse - 5′CACCACCACCCACGGAATCG3′

### Migration assay

Primary HSC at day 8 were trypsinized and plated within the cell culture inserts (Cat no# 80209, ibidi, Munich, Germany) placed in a 24 well plate (0.25 x 10^6^/chamber). When cells formed confluent tight monolayer, they were serum starved for 24 h to arrest them in G_0_ phase_._ Inserts were then carefully removed, resulting in a gap of approximately 500 nm between the two monolayers. Cells were washed twice with sterile PBS and then cultured in DMEM media (10% heat inactivated FBS, 1% PSF) with or without recombinant mouse C5a (10 ng/mL), mouse PDGF-BB (PDGF) (50 ng/mL), or PDGF and C5a. This concentration of C5a was chosen based on physiologically relevant concentrations observed in liver [27,18] and preliminary experiments testing a range of concentrations from 5 to 30 ng/mL C5a. Maximal stimulation of migration was observed at 10 ng/mL (data not shown). In the experiment with CCR2 (MCP-1 receptor) antagonist (227016, Millipore), 227016 (10 nM) was added to the cell media 30 min prior to treatment with PDGF or C5a. Images were captured using Olympus IX81 Microscope at 0, 10, and 24 h. Gap width and rate of wound healing was analyzed using Wimasis Image analysis software (Munich, Germany).

### Boyden chamber chemotaxis assay

Boyden chambers were used to assess HSC chemotaxis using the QCM 5 um Chemotaxis assay kit from Millipore (Cat # ECM506), per manufacturer instructions. Briefly, primary HSC at day 8 were serum-starved in DMEM with 0.5% FBS for 18 h. HSCs were then washed twice with 1X PBS and detached using Accutase cell detachment solution (BD Pharmingen). Cells were counted and suspended in serum-free DMEM (0.3 × 10^6^/mL) and 250 uL of the cell suspension added into each insert. A total of 500 uL of serum-free DMEM, with or without C5a or PDGF, was then added to the lower chamber. After incubation at 37°C for 10 h, inserts were stained, washed, and destained and optical density measured at 560 nm. Cells from the upper membrane layer were removed prior to destaining in order to decrease non-specific background staining.

### Flow cytometry

To study HSC activated *in vivo*, transgenic mice with GFP tagged to the collagen promoter were injected with carbon tetrachloride (CCl_4_) for 5 weeks (two injection/week, one dose 0.25 μL/g body weight, one dose of 0.5 μL/g body weight, and eight doses of 1.0 μL/g body weight). Total non-parenchymal cells (NPC) were isolated and immediately used for staining. Cells were centrifuged at 800Xg for 4 min; pellet was washed with 1X PBS and re-suspended in 50 μL of FACS buffer (1% BSA, 0.1% sodium azide in 1X PBS). Cells were blocked with 1.0 μg of anti-mouse CD32/CD16 FCγ receptor blocking antibody for 15 min at 4°C. Cells were then stained with 1.5 μg C5aR (CD88) and 0.5 μg C5L2 phycoerythrin-conjugated primary antibody or isotype control (phycoerythrin-conjugated IgG1) diluted in FACS buffer and for 30 min at 4°C. Cells were centrifuged at 800Xg for 4 min, washed once with FACS buffer, and re-suspended in 50 μL fixing buffer (1% paraformaldehyde in 1X PBS). Stained cells are kept in the dark at 4°C overnight, following which they were centrifuged at 830 × g for 4 min. Stained cells were suspended in 300 uL of FACS buffer, and data were collected on a LSRII flow cytometer (Becton Dickinson Immunocytometry systems, Mountain View, CA, USA). Data collected on the LSRII were analysed using FlowJo software (Tree Star, Inc., Ashland, OR, USA).

### Immunocytochemistry

Primary HSC (day 5 and day 10 of culture) were detached from their growth plates and re-seeded in an 8-well chamber slide (Nagle Nunc International, Rochester, NY) at a density of 1.0 × 10^6^ cells/mL, 0.5 mL/well. After overnight incubation (to allow complete attachment of cells and recovery from trypsinization), cells were washed with ice-cold 1X PBS and fixed with 4% paraformaldehyde for 10 min at room temperature (0.5 mL/well). Cells were washed with 25 mM glycine (two times, 1 min each, 0.5 mL/well), followed by two washes in PBS (1 min each, 0.5 mL/well). Cells were blocked with 1X PBS containing 1% fish gelatin containing 2% BSA and 0.1% Triton-X-100 for 1 h and incubated overnight with polyclonal mouse C5aR antibody (diluted 1:25 in blocking buffer without Triton-X) at 4°C in a humidified chamber. After three washes in PBS buffer (3 times 10 min each), cells were incubated with the fluorochrome-conjugated secondary antibody (Alexa fluor 488 labelled goat-anti-rabbit IgG, 1:50 diluted in blocking buffer without Triton X) for 1 h at room temperature. Cells were then washed three times in PBS and mounted with VECTASHIELD 1200 (with DAPI). A slide without primary antibody was used as negative control. Fluorescence images were acquired using a LEICA DMRXE confocal microscope.

### Statistical analysis

Values are reported as means ± standard error of the means (SEM). Data were analyzed by analysis of variance (ANOVA) using general linear models procedure (SAS, Cary, NC, USA). Multiple comparisons were analysed using least square means.

## Abbreviations

α-SMA: Alpha smooth muscle actin; C5a: Complement component 5a; C5aR: C5a receptor; CCl_4_: Carbon tetrachloride; CCR2: MCP-1 receptor; Col1A1: Collagen 1A; GFP: Green fluorescent protein; GPCR: G-protein coupled receptor; HBSS: Hank’s balanced salt solution; HSC: Hepatic stellate cells; MCP-1: Monocyte chemoattractant protein-1; NPC: Non-parenchymal cells; PDGF: Platelet derived growth factor-BB.

## Competing interests

The authors declare that they have no competing interests.

## Authors’ contributions

LEN conceived the study. LEN and DD designed and executed the experiments. DD optimized and performed HSC isolation, migration assay, western blot, flow cytometry, and qRT-PCR. MAB assisted in the design, execution, and data analysis for the flow cytometry. LEN and DD contributed to the writing of the paper and organizing the figures. All authors approved the final version.
